# A Novel Nano-approach for Targeted Inner Ear Imaging

**DOI:** 10.4172/2157-7439.1000456

**Published:** 2017-08-31

**Authors:** MN Kayyali, L Brake, AJ Ramsey, AC Wright, BW O’Malley, D Daqing Li

**Affiliations:** 1 Department of Otolaryngology, Head & Neck Surgery, University of Pennsylvania Health System, Philadelphia, USA; 2 Department of Radiology, University of Pennsylvania, USA

**Keywords:** Inner ear imaging, Targeted contrast agents, Gold nanoparticles, Liposomal iodine

## Abstract

During the last decade, there have been major improvements in imaging modalities and the development of molecular imaging in general. However detailed inner ear imaging still provides very limited information to physicians. This is unsatisfactory as sensorineural hearing loss is the main cause of permanent hearing loss in adults and at least 134 genetic mutations that result in congenital hearing loss have been identified. We are still unable, in most cases where gross anatomical changes are not observed, to determine the exact cause of hearing loss at a cellular or molecular level in patients using non-invasive techniques. This limitation in inner ear diagnostic modalities is a major obstacle behind the delay in discovering treatments for many of the causes of sensorineural hearing loss. This paper initially investigated the use of targeted gold nanoparticles as contrast agents for inner ear imaging. These nanoparticles have many useful characteristics such as being easy to target and possessing minimal cytotoxicity. We were able to detect the nanoparticles diffusing in the hair cells using confocal microscopy. Regrettably, despite their many admirable characteristics, the gold nanoparticles were unable to significantly enhance CT imaging of the inner ear. Consequently, we investigated liposomal iodine as a potential solution for the unsatisfactory CT contrast obtained with the gold nanoparticles. Fortunately, significant enhancement of the micro-CT image was observed with either Lugol’s solution or liposomal iodine, with Lugol’s solution enabling fine inner ear structures to be detected.

## Introduction

Hearing loss (HL) is one of the world’s most common disabilities, with World Health Organization estimating in 2008 that over 360 million people worldwide suffered from disabling HL [1]. HL in infants often has a genetic cause, with at least 134 genes being associated with HL [2,3]. The pathophysiology and pathologies that underlie sensorineural HL are highly varied, ranging from major structural malformations of the inner ear [4], to caspase induced apoptosis of hair cells [5] and to the mutation of a key protein such as cadherin-23 [6]. Audiological tests are able to quantify HL without specifying the underlying pathology. This is because, in many cases, the pathophysiology of HL occurs at the cellular and subcellular levels. This makes it difficult to determine the underlying causes using the currently available techniques. The anatomical structure, along with the delicate and sensitive nature of the inner ear, precludes the use of invasive techniques such as biopsy, which may result in complete auditory and vestibular loss. Therefore a non-invasive technique is required to determine the etiology of sensorineural HL.

Imaging techniques, such as magnetic resonance imaging (MRI) and X-ray based computed tomography (CT), are routinely utilized in an otorhinolaryngology clinical setting. CT images the temporal bone and other osseous structures in great detail, but it is limited in visualizing soft tissue [7]. Conversely, while MRI creates high quality images of soft tissues [8], it produces low resolution images of the bony landmarks of the inner ear. Artifacts arising from the air/bone interface encountered in the temporal bone further compromise MRI. One possible method of overcoming these problems is the use of contrast agents [9,10]. Traditional contrast agents are employed to differentially increase or decrease the signal observed in the tissue of interest. The contrast agent’s specificity and sensitivity can be enhanced by conjugating them to a targeting group. These groups allow the contrast agents to bind to specific structures and produce localized areas of high contrast that can be readily detected on the resulting image [10].

Intravenously delivered, untargeted gadolinium contrast agents are commonly used to enhance MRI imaging of the inner ear. These agents have enabled endolymphatic hydrops associated with Meniere’s disease to be detected [11,12]. Unfortunately, attempts to use iron oxide micelles as inner ear MRI contrast agents have been met with limited success [13]. Surprisingly, there are no reports of enhancement of MR imaging of the inner ear using targeted contrast agents. The use of contrast agents in CT imaging typically produces much lower image enhancement than with MRI [14]. In addition, the anatomical structure of the inner ear makes it difficult to load sufficient amounts of a contrast agent into the inner ear for CT image enhancement to occur. Consequently, CT imaging of the inner ear is currently routinely performed without contrast agents [15–17].

This study considered 2 potential contrast agents for CT imaging: gold nanoparticles (GNPs) and iodine-based agents. GNPs possess a range of desirable physical attributes that make them good candidates to be inner ear contrast agents [18]. These properties include high biocompatibility [19,20], insignificant cytotoxicity [21] and the ability to readily conjugate to specific targeting biomarkers [22]. CT imaging in other tissues has demonstrated that targeted GNPs greatly increase CT image sensitivity [23] and enable the visualization of small structures [24]. These attributes are essential in inner ear CT imaging.

Iodine-based contrast agents, which are already widely used in a clinical setting [7], were identified as alternate candidates for enhancing CT images of the inner ear. These compounds are much less expensive than their gold counterparts and have been shown to produce a higher degree of signal attenuation than the GNPs under clinical CT imaging protocols [21]. The current generation of iodinated contrast agents possess minimal cytotoxicity and have been designed to minimize non-specific binding [10].

To further enhance the image quality, the contrast agents can be targeted to specific cell types. The 12-mer peptide, A665 binds selectively to the extracellular loops of prestin [25], a transmembrane motor protein which plays an essential role in sound transduction and is specifically expressed in outer hair cells (OHCs) [26]. In addition, mutations of this protein are responsible for congenital forms of deafness [27], and OHC apoptosis is one of the most common causes of HL [28]. Consequently, contrast agents targeted with a prestin binding peptide have great potential both as CT image enhancers and as diagnostic tools. The House Ear Institute-Organ of Corti 1 (HEI-OC1) cell line was chosen for *in vitro* studies because it expresses many inner ear biomarkers, including prestin which mainly expresses in the cellular outer membrane under non-permissive conditions [29]. While GNPs are readily conjugated to targeting agents, it is comparatively difficult to conjugate the targeting compounds onto iodinated contrast agents[10]. Consequently, this study initially focused on targeting GNPs to HEI-OC1 cells and OHCs. It also laid the foundation for targeted iodine-based agents by fabricating iodine rich liposomes that are suitable for conjugation to molecules that target a specific biomarker [30].

Finally, because of poor blood perfusion and the blood-labyrinth barrier, the contrast agents were delivered locally. Transtympanic injection is the most common means to locally deliver agents into the inner ear [31]. This technique, however, is inefficient as the agent can readily exit the middle ear *via* the Eustachian tube. In this study, the contrast agents were delivered by injecting them into the round window niche, which was then sealed by the placement of chitosan hydrogel mixed with the appropriate contrast agent [32]. Once the hydrogel had been applied, it warmed to body temperature and transformed from a liquid to a semisolid form [33]. This constrained the contrast agent to the round window membrane, allowing the particles to diffuse into the cochlea [34].

## Materials and Methods

### Materials

Both the unconjugated and A665-conjugated GNPs were fabricated by the i-colloid division of IMRA America Inc., Ann Arbor MI. The A665 peptide was fabricated by Genscript Biotech Corporation, Piscataway NJ. The rabbit anti-prestin and Alexa 488-conjugated goat anti-rabbit IgG antibodies were purchased from Santa Cruz Biotechnology Inc., Dallas TX. Remel^™^ Lugol’s Iodine was obtained from Thermo Fisher Scientific and the chitosan 95/1000 was purchased from Heppe Medical Chitosan GmbH, Halle, Germany. The iodinated contrast agents Lipiodol and Iopamidol were manufactured by Guerbet Pharmaceutical Company, Bloomington IN and Bracco Diagnostics, Monroe Township NJ, respectively.

### Cell culture

The cells were grown in high-glucose Dulbecco’s Modified Eagle’s Medium (DMEM) supplemented with 10% fetal bovine serum and 1% penicillin. HEI-OC1 cells were propagated in permissive conditions of 33°C and 10% CO_2_ [29]. When it was necessary to maximize the levels of prestin in the outer membrane, the cells were grown under non permissive conditions of 39°C and 5% CO_2_ [29] where cells will be able to differentiate and prestin expression will be upregulated.

### Gold nanoparticle fabrication and conjugation

Spherical GNPs with a peak diameter of 50 nm were synthesized (i-colloid division, IMRA America Inc., Ann Arbor MI). When requested, the GNPs were conjugated and characterized by the company to the A665 12 mer peptide. Conjugation was confirmed using dynamic light scattering analysis where a 2–3 nm increase in the GNPs’ hydrodynamic size was observed upon peptide conjugation.

### Liposomal iodine

Liposomes containing Liopamidol and Lipiodol were fabricated using the reverse phase evaporation protocol of Kweon et al. [35]. The resulting liposomes were concentrated using Amicon®Ultra 10 K centrifuge filters and stored at 4°C until used.

### MTT cell viability assays

1000 HEI-OC1 cells were incubated overnight under permissive conditions before sterilized GNPs were added to the cells. The cells were then incubated under permissive conditions for up to 6 days without changing the media. The Vybrant® MTT Cell Proliferation Assay protocol was followed, with the minor modification that the cells were incubated with the SDS solution for only 90 minutes.

### Dark field imaging

A modified version of the protocol of Huang et al. [36] was utilized to produce dark field images. The HEI-OC1 cells were grown under non-permissive conditions in order to allow the cells to differentiate. Then GNPs (0.25 mg/ml) in DMEM were added to the cells for 24 hours to allow the nanoparticles to bind. Dark field microscopy images were then taken using a Nikon Eclipse T5 100 inverted microscope.

### Hydrogel preparation

A 2% w/v solution of chitosan 95/1000 in 0.1 M HCl was prepared by stirring overnight at room temperature and then stored at 4°C until required. The chitosan hydrogel was then prepared as previously described [37]. Once the viscous hydrogel sample had been prepared, GNPs or liposomal iodine were added to the hydrogel in a 1:1 ratio (v/v), with stirring, until a homogenous nanohydrogel formulation was obtained. The hydrogel samples were kept on ice until used, within 2 hours of preparation.

### *In Vivo* assessment of contrast agents

All animal care and use was in accordance with the instructions from the Institutional Animal Care and Use Committee of The University of Pennsylvania. Mice were anesthetized using an intraperitoneal injection of a Ketamine/Xylazine (100/10) cocktail. The nanohydrogel samples were then applied to round window niches after direct injection into the inner ear as previously described [38].

### Euthanasia and cochlear dissection

Twenty-four hours after surgery, mice were euthanized using cervical spine dislocation following deep anesthesia with an intraperitoneal injection of a Ketamine/Xylazine (100/10) cocktail. The dissected cochleae were either scanned using a micro-CT imaging system (Scanco μCT 35) or sent to Dr Rende Gu (Seattle WA) for tissue harvesting. The cochleae that had been exposed to fluorescein-5-Isothiocyanate (FITC) bound GNPs were imaged by micro-CT immediately. However, cochlear tissue obtained for morphological studies was exposed to rabbit anti-prestin antibodies (Santa Cruz) followed by Alexa 488 conjugated goat anti-rabbit IgG (Santa Cruz). This cochlear tissue was then imaged by confocal microscopy.

### Embryonic cochlear explant studies

P6 mouse cochleae were dissected and cultured using DMEM: Nutrient Mixture F-12 (Life Technologies, Carlsbad, CA) supplemented with 10% fetal bovine serum, 1% penicillin and 1% flucytosine and incubated at 37C with 5% CO_2_. After 24 hours, 10% by volume of either A665 targeted or untargeted GNPs, both containing FITC, were added to the explants and allowed to interact overnight. The tissue was then fixed and the presence of fluorescent gold particles was monitored by confocal microscopy.

### *Ex Vivo* experiments

The mice were euthanized and decapitated before their heads were sterilized with 70% ethanol. The cochleae were removed and the round window membrane punctured using a fine-tipped syringe before being fixed overnight with 4% paraformaldehyde at 4°C. The cochleae were then injected with either Lugol’s iodine or liposomal iodine.

### Micro-CT imaging

The cochleae were soaked in PBS before being scanned in an ultra-high resolution specimen micro-CT imaging system (μCT 35, Scanco USA Inc., Wayne PA), typically using the following parameters: 55 kV, 145 μA, 6 micron isotropic resolution, 2 × 2 binning, 800 ms integration time. The images were then analyzed using Osirix software.

## Results

### GNP toxicity

**Figure 1:**
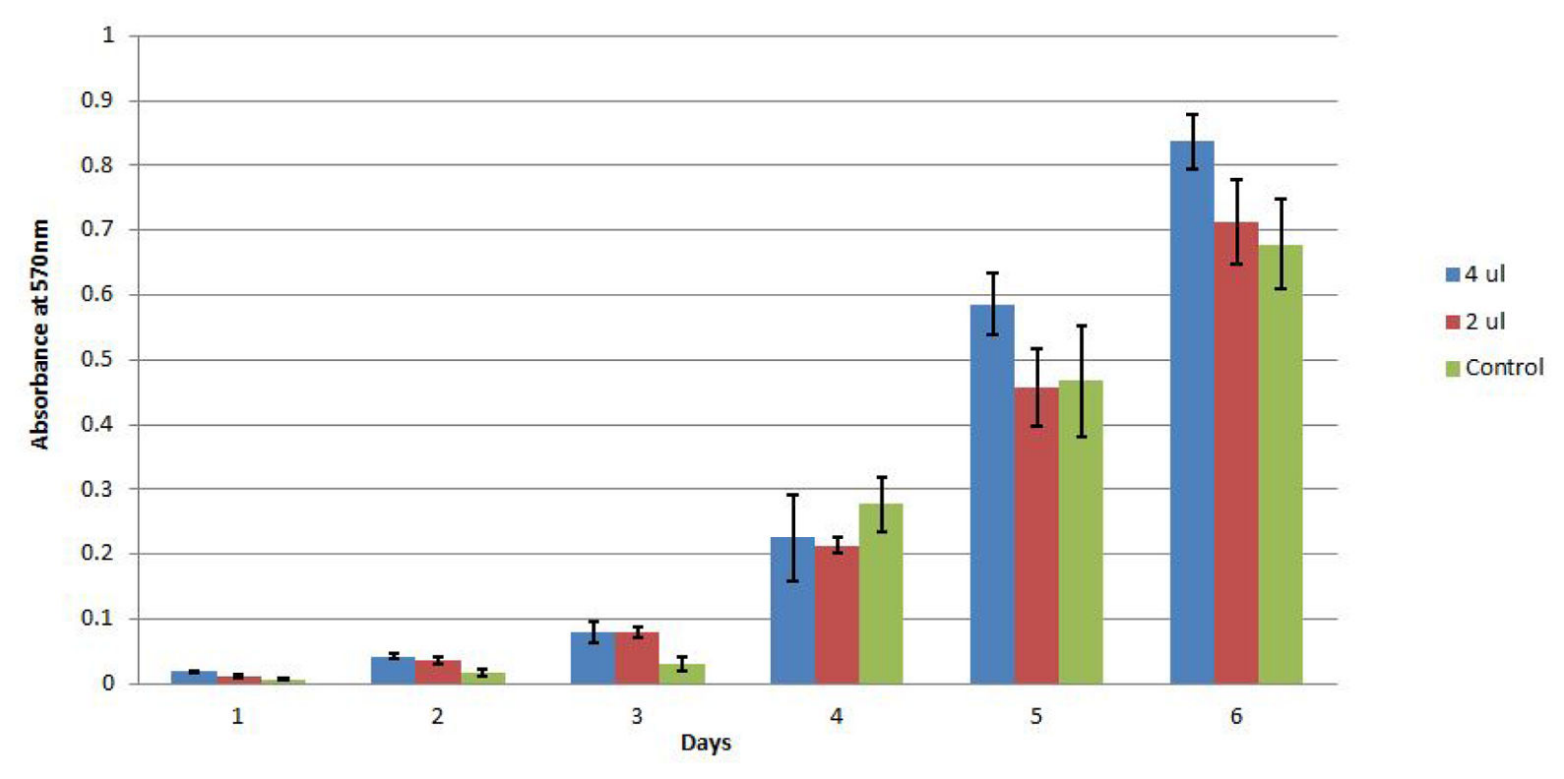
Absorbance values for HEI-OC1 cells on a 6 day MTT time-course after exposure to GNPs. HEI-OC1 cells were incubated with three concentrations of GNPs: 0 (Green), 50 μM (red) and 100 μM (Blue) for up to 6 days. The Vybrant^®^ MTT cell proliferation assay protocol was followed. This procedure was conducted in triplicate and demonstrated that GNPs have a minimal effect on cell viability.

**Figure 2:**
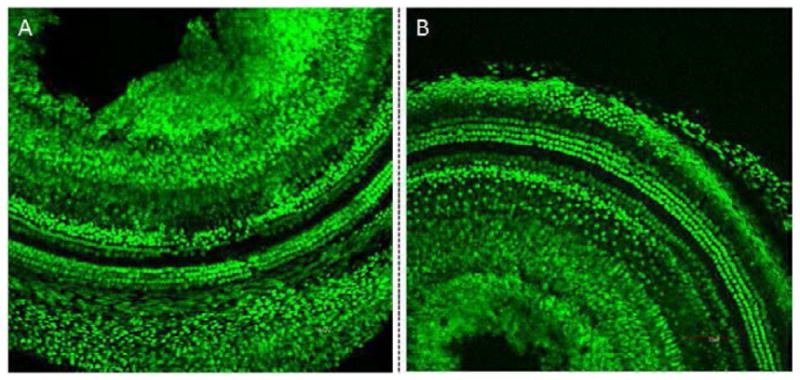
Confocal microscopy images of mouse cochleae following treatment with GNPs (A) and the untreated control (B). GNPs were surgically applied into the mouse cochlea. After 24 hours, the cochleae were fixed and stained with a prestin-488 antibody. The results indicate that no significant difference in the OHC morphology was observed between cochleae that had been exposed to GNPs and the control cochleae. This strongly suggests that GNPs do not cause significant morphological changes.

The GNPs were characterized to ensure that they were suitable for clinical use. Initially, the sensitivity of the mouse cochlear cell line HEI-OC1 to the GNPs was investigated using the MTT cell viability assay. No significant changes in cell viability were observed following the exposure of the cells to differing concentrations of GNPS for 6 days (Figure 1). The effect of the GNPs on hair cell morphology was then evaluated by applying the GNPs to mouse cochleae *in vivo*. Twenty four hours later, the cochleae were fixed and stained with a prestin-488 antibody. The resulting cochlear images (Figure 2) demonstrate that the presence of the GNPs had no observable effect on the morphology of the hair cells.

### Specificity of targeted gnp binding

**Figure 3:**
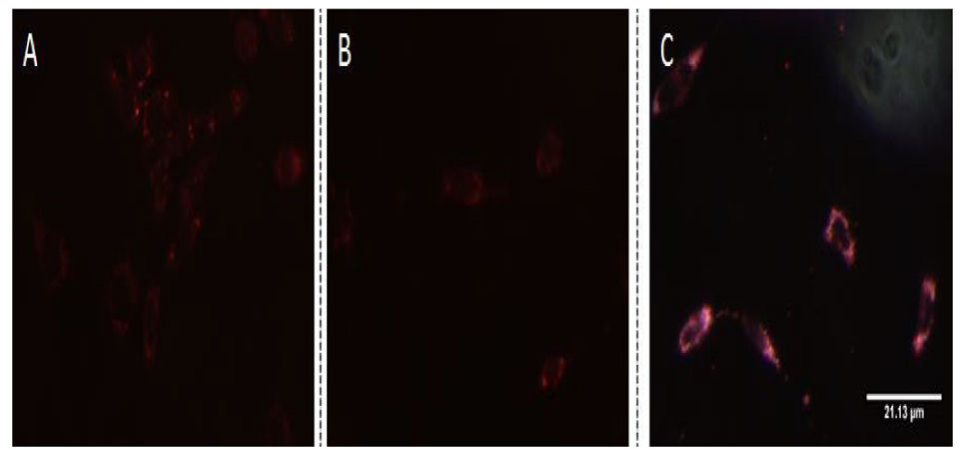
Dark field microscopy images of HEI-OC1 cells. Targeted GNPs (0.25 mg/ml) (C) or untargeted GNPs (0.25 mg/ml) (B) were added to differentiated HEI-OC1 cells. The treated cells were then incubated for 24 hours, alongside control HEI-OC1 cells where GNPs were not added (A), to allow the nanoparticles to bind to the cells. The dark field images clearly show that much greater light scattering was observed with targeted GNPs (C) than with the other samples. This strongly suggests that the GNPs bound specifically to the HEI-OC1 cells: presumably to prestin.

**Figure 4:**
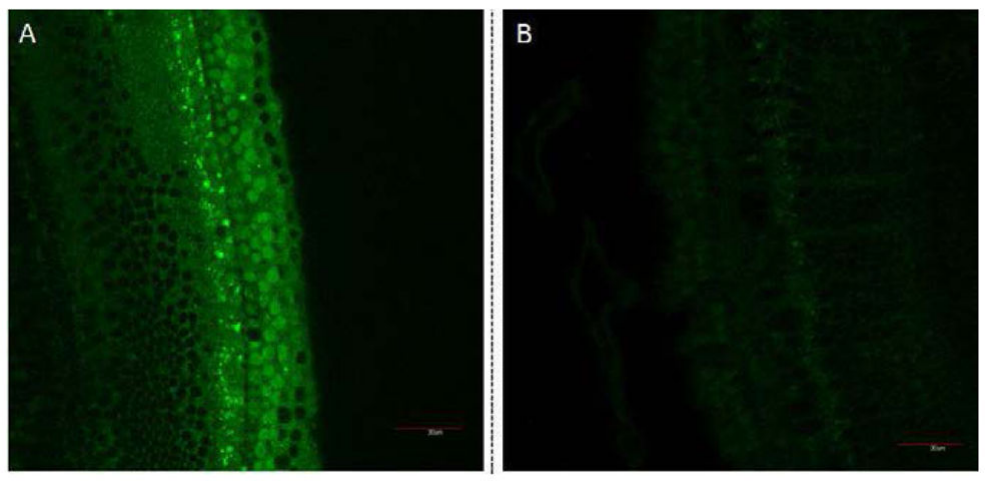
Confocal microscopy images of cochlear organotypic cultures incubated with targeted GNPs (A), untargeted GNPs (B). Fluorescent GNPs (0.25 mg/ml) were added to cochlear organotypic cultures and the samples incubated for 24 hours to allow the nanoparticles to bind to the cells. The images clearly indicate that the targeted GNPs were binding specifically to the outer hair cells.

The ability of the targeted GNPs to bind specifically to prestin was first evaluated in HEI-OC1 cells using dark field imaging [39]. Figure 3 illustrates that greater light scattering was observed around cells in the targeted GNPs sample (3C) than in the untargeted GNPs (3B) or the no GNPs (3A) control samples. This suggests that the targeted GNPs bind specifically to the prestin expressed by the HEI-OC1 cells [29]. Cochlear organotypic cultures were then exposed to FITC-labeled targeted and untargeted GNPs. Figure 4 demonstrates that very limited fluorescence was observed in the cochlear sample whose hair cells had been exposed to untargeted GNPs. However, incubation of the cochlea with the targeted GNPs resulted in very strong fluorescence signals being observed around the hair cells. This demonstrates that the presence of the A665 peptide enabled the targeted GNPs to bind specifically to the OHCs, presumably to prestin.

### CT imaging using gold nanoparticles

**Figure 5:**
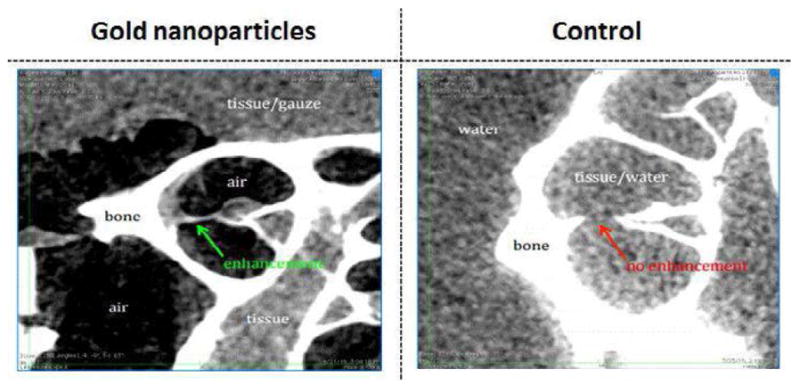
The use of gold nanoparticles to enhance CT imaging of the inner ear. GNPs were applied *in vivo* to mouse cochleae. The resulting CT images suggest that the presence of GNPs produced very limited enhancement of the CT image (green arrow), as compared to the tissue signal from the control sample where GNPs had not been added (red arrow).

**Figure 6:**
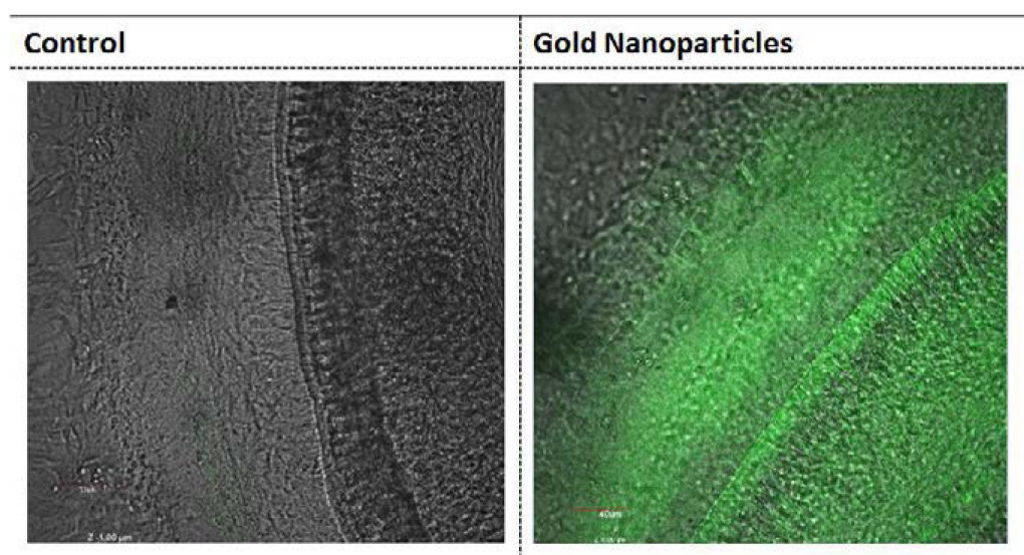
Detection of gold nanoparticles inferred using confocal microscopy. GNPs bound to FITC were applied to mouse cochlea *in vivo*. After 24 hours, the cochleae were dissected and imaged under confocal microscopy. Fluorescence was observed throughout the cochlea that had been exposed to GNPs (B) while little fluorescence was detected in control cochlea where GNPs had not been added (A). This suggests that the GNPs injected into the mouse cochlear had fully diffused throughout the inner ear (B).

The ability of GNPs to enhance CT imaging was evaluated by scanning cochlea using the Scanco *μCT* 35 micro-CT system. Figure 5 (left) demonstrates that the GNPs, even at high concentrations (2.5 mg/ml), produced virtually no enhancement of the CT images of the Organ of Corti. However, Figure 6 illustrates that sufficient fluorescent GNPs were delivered to mouse cochlea *in vivo* that they could be detected by confocal microscopy. This proves that although no enhancement of the CT image was observed, the GNPs had been successfully localized within the cells. Taken together, the two experiments demonstrate that GNPs do not noticeably enhance X-ray attenuation and therefore are not suitable for enhancing CT imaging of the inner ear.

### CT imaging using iodinated contrast agents

**Figure 7:**
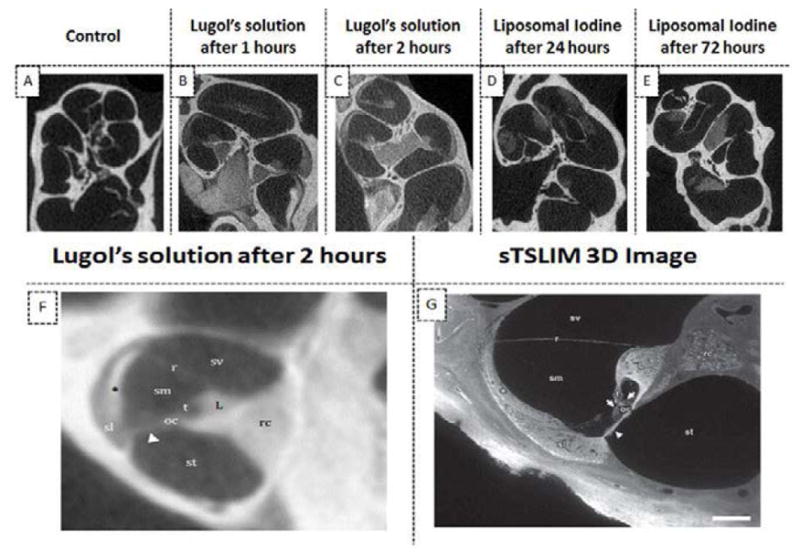
The use of iodine based contrast agents to enhance micro-CT images of mice cochleae. The *ex vivo* administration of Lugol’s iodine to cochleae produced significantly higher image signal and contrast at all time points than in images of untreated cochleae (A–C). In particular, there was a marked improvement in the imaging of the Organ of Corti (B and C). Most of the fine cochlear structures were observed in cochlear images taken after 2 hours incubation (F). The detail observable in the micro-CT image of (F) is comparable to that of a sTSLIM 3D optical image[40] (G): scala vestibuli (sv), media (sm) and tympani (st); basilar (arrowhead), tectorial (t) and Reissner’s membrane (r); stria vascularis (asterisk), spiral ligament (sl), organ of Corti (oc) with hair cells (arrows), spiral limbus (L) and Rosenthal’s canal (rc). The presence of liposomal iodine (LI) also enhanced the micro-CT images of the cochleae (D–E). Note: The micro-CT image in panel F was acquired at 12 micron resolution and is an average of 10 contiguous slices through a 120 micron thick slab.

Micro-CT images of explants stained with Lugol’s solution for either 1 or two hours showed significant enhancement of the soft tissue signal (Figure 7A–7C). In particular, there was a marked improvement in image signal and contrast in the area of the Organ of Corti, with most of the fine cochlear structures being visible after only 2 hours of incubation with the contrast agent (Figure 7F).

**Figure 8:**
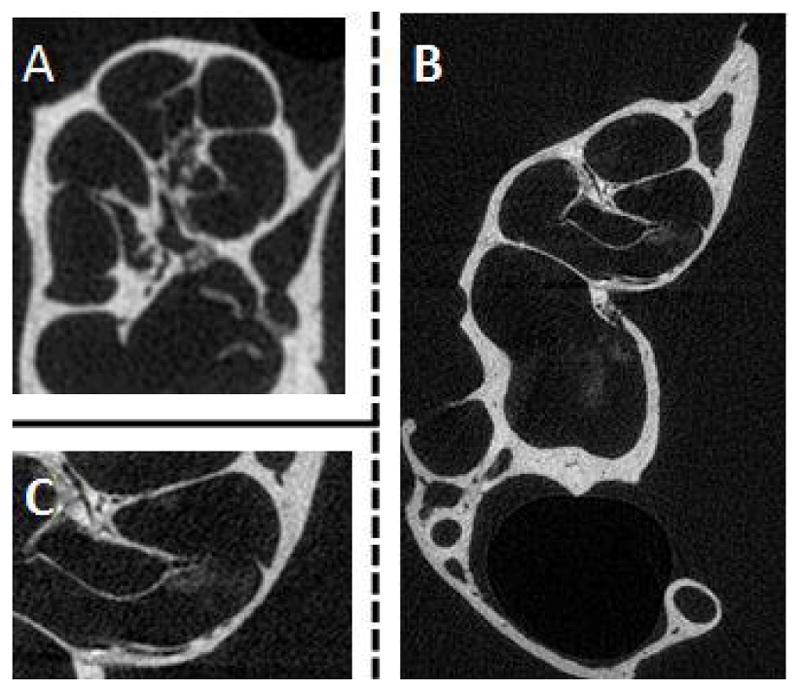
Detection of liposomal iodine in cochlear CT imaging after *in vivo* application of liposomal iodine. Liposomal iodine was applied *in vivo* into mouse cochleae. After dissection, the micro-CT imaging system was used to scan the untreated control (A) and liposomal iodine-treated (B) cochleae. The magnified image of the liposomal iodine-treated cochlea (C) clearly shows the presence of the liposomal iodine, which had collected near the Organ of Corti. This was not detected in the control group (A). Although the liposomal iodine was detected inside the test cochlea, it did not enter the cells in sufficient quantities to enhance CT soft tissue imaging.

Figure 7D and 7E demonstrate that the *ex vivo* application of liposomal iodine to cochleae also enhanced micro-CT imaging. Micro-CT image enhancement was also observed following the *in vivo* application of liposomal iodine to mouse cochleae (Figure 8B and 8C). Unfortunately, while the liposomal iodine appeared to be able to diffuse throughout the cochlear fluids, it was unable to enter the cochlear cells in sufficient quantities to enhance micro-CT soft tissue imaging.

## Discussion

There is a great demand in the otorhinolaryngology clinic for a non-invasive diagnostic modality to detect the histopathology in the inner ear at the cellular and subcellular levels. Unfortunately, major obstacles exist when using the two most common non-invasive diagnostic techniques, CT and MRI, to image the inner ear. Although CT images the temporal bone in exquisite detail, the signal-to-noise ratio and image contrast observed in the inner ear soft tissue is insufficient for clinical needs. This paper describes an attempt to overcome the shortcomings of CT imaging by using contrast agents: GNPs and iodinated contrast agents. These nanoparticles can attenuate X-rays, but in order to have more diagnostic value within the inner ear, the contrast agent has to be targeted to specific cell types. We utilized the prestin binding peptide A665 [25] to enable the GNPs to bind to the cochlear OHCs, creating an optically detectable localized high concentration of GNPs.

It was critical to confirm that GNPs were non-toxic for potential clinical applications. MTT assays demonstrated that the nanoparticles were not cytotoxic (Figure 1). Furthermore, the addition of the GNPs to the cochlea did not produce any morphological changes (Figure 2). Taken together, the two techniques demonstrated that the GNPs are not toxic to the inner ear at the concentrations used in the assays.

The ability to effectively target GNPs to specific cells types was investigated by studying the ability of A665-conjugated GNPs to bind to prestin in the membranes of HEI-OC1 cells and OHCs. Dark field microscopy and confocal microscopy demonstrated that the targeted GNP constructs were able to bind strongly to prestin expressing HEI-OC1 cells (Figure 3) and that the FITC-conjugated targeted GNPs bound to OHCs in cochlear organotypic cultures (Figure 4A). Consequently, the two experiments strongly suggest that the A665-conjugated GNPs are capable of targeting prestin on hair cells.

Despite the GNPs possessing a number of desirable properties, the observed enhancement of CT imaging by the particles was minimal. One possible solution to this problem is to increase the concentration and volume of GNPs. However, this will be difficult due to the complex, enclosed structure of the cochlea, the high sensitivity of inner ear tissue to foreign agents and the potential for increased non-specific binding.

In the clinic, iodine based compounds are the most popular CT contrast agents. They have many advantages over gold based contrast agents including FDA approval, lower cost, wide availability and the ability to produce higher X-ray attenuations under clinical CT protocols. Lugol’s solution consists of a simple aqueous mixture of elemental iodine and potassium iodide. The *ex-vivo* injection of Lugol’s solution into cochleae produced differential tissue contrast which greatly enhanced the resulting CT image. The use of Lugol’s solution enabled detailed images of the inner ear that were comparable to a 3D image obtained using sTSLIM [40]. The ability of liposomal iodine to enhance CT imaging was then evaluated. In addition to the inherent advantages of iodine-based contrast agents, liposomal iodine is readily targeted [30]. This study used liposomes composed of water soluble Lopamidol encapsulated in a lipid bilayer containing Lipidol - an iodinated derivative of poppy seed oil. The combination of the water soluble and lipid soluble iodinated contrast agents was used to maximize the contrast observed during CT scanning. We intend to synthesize A665 bound liposomal iodine constructs which will enable the construct to specifically bind to, and enhance CT imaging of, OHCs. Fortunately, both the ex *vivo* and *in vivo* addition of liposomal iodine to the mouse cochlea enhanced CT imaging. Regrettably, the liposomes did not diffuse inside the cells as well as the Lugol’s solution. Clearly the iodinated liposomes need to be optimized. In particular, their size will have to be adjusted to improve their uptake by cochlear cells. The construct’s lipid coat will also have to be modified in order to optimize the liposomes’ solubility within the perilymph.

This study clearly demonstrates that GNP contrast agents are not suitable for inner ear applications. Liposomal iodine however shows the potential to generate high quality CT images of the inner ear. We are working to improve the physical characteristics of the liposomal iodine and, additionally, the targeting of the liposomal iodine to the OHC-specific prestin biomarker. These goals seem to be readily achievable and the use of targeted CT contrast agents may solve the inner ear imaging challenges that currently are encountered in the clinical setting.
